# Sub- and Supra-Second Timing in Auditory Perception: Evidence for Cross-Domain Relationships

**DOI:** 10.3389/fnins.2021.812533

**Published:** 2022-01-12

**Authors:** Elzbieta Szelag, Magdalena Stanczyk, Aneta Szymaszek

**Affiliations:** Laboratory of Neuropsychology, Nencki Institute of Experimental Biology of the Polish Academy of Sciences, Warsaw, Poland

**Keywords:** auditory perception, temporal information processing, timing, sub-second timing, supra-second timing

## Abstract

Previous studies indicate that there are at least two levels of temporal processing: the sub- and supra-second domains. The relationship between these domains remains unclear. The aim of this study was to test whether performance on the sub-second level is related to that on the supra-second one, or whether these two domains operate independently. Participants were 118 healthy adults (mean age = 23 years). The sub-second level was studied with a temporal-order judgment task and indexed by the Temporal Order Threshold (TOT), on which lower values corresponded to better performance. On the basis of TOT results, the initial sample was classified into two groups characterized by either higher temporal efficiency (HTE) or lower temporal efficiency (LTE). Next, the efficiency of performance on the supra-second level was studied in these two groups using the subjective accentuation task, in which participants listened to monotonous sequences of beats and were asked to mentally accentuate every n-th beat to create individual rhythmic patterns. The extent of temporal integration was assessed on the basis of the number of beats being united and better performance corresponded to longer units. The novel results are differences between groups in this temporal integration. The HTE group integrated beats in significantly longer units than did the LTE group. Moreover, for tasks with higher mental load, the HTE group relied more on a constant time strategy, whereas the LTE group relied more on mental counting, probably because of less efficient temporal integration. These findings provide insight into associations between sub- and supra-second levels of processing and point to a common time keeping system, which is active independently of temporal domain.

## Introduction

### Temporal Constraints of Cognitive Functions

Time may be considered as the frontier in cognitive sciences and a fundamental property of working human brains. Much evidence from both everyday observations and extensive research studies has consistently indicated that many cognitive functions—such as language, perception, short-term and working memory, attention, motor activity, decision making, executive functions, etc.,—are temporally segmented in specific time intervals and are rooted in a defined temporal template (e.g., Szelag et al., 2004a,^2008^, ^2010^, ^2011^, ^2014^; Szymaszek et al., 2009, 2018; Ulbrich et al., 2009; Oron et al., 2015; Nowak et al., 2016; Buhusi et al., 2018; Szelag, 2018; Choinski et al., 2020; Jablonska et al., 2020). Temporal information processing (TIP) is omnipresent, for example in every verbal or perceptual act, in movement control, in learning, and in planning. Efficient intrinsic timing mechanisms are necessary to effectively execute these activities. Patterning in time, therefore, is considered as providing a structure for complex cognition. Because of such ubiquity of time in mind, the basic question arises: how is temporal information processed in our brains?

As the first step toward answering this question, we can refer to the taxonomy system of our time experiences. Based on the data from a variety of studies, we can postulate that temporal perception is not veridical and subjective time is not a linear function of clock time. Deviations from the timed template have been commonly observed in psychological studies. Moreover, a large body of experimental studies has consistently indicated that humans can process temporal information over several time scales, or operational processing windows, which may be categorized into major groups (Pöppel, 1994; Mauk and Buonomano, 2004).

According to the framework model of TIP proposed by Pöppel (1997, 2004, 2009); see also Wittmann, 2009, 2013; Montemayor and Wittmann, 2014), one can distinguish a few hierarchically ordered timescales controlling our mental operations. This model offers new ways to explain different temporal phenomena, such as perception of order (corresponding to temporal resolution), the feeling of “nowness,” and so on. Of crucial importance in this taxonomy system are two distinct domains: one operating in a range of some tens of milliseconds and the other in a range of a few seconds. These two domains are involved in different mental processes and may be studied with different experimental paradigms. Hence, they seem to be controlled by different neuronal mechanisms.

It should be stressed that in this paper we do not refer to any “constant numerical values” obtained in particular experimental studies, but rather indicate two temporal domains or operational processing windows: one of sub-seconds, the other of supra-seconds with, of course, intra- and inter-individual variability.

### Hierarchical Multi-Timescale Framework Model for Cognitive Functions

#### Sub-Second Processing and Temporal Resolution

The sub-second level is related to perception of succession and identification of the temporal order of incoming events. Mental operations at this processing level are rooted in temporal resolution—the ability to perceive the order of stimuli presented in rapid sequences, thus, the identification of their before–after relation. Such temporal resolution requires, first, the identification of particular incoming stimuli within a sequence and, then, the perception of their order. This enables efficient flow of cognitive processes and coordination of every perceptual or motor act (Buhusi and Meck, 2005; Fostick and Babkoff, 2013, 2017; Wittmann, 2013). The efficiency of a subject’s performance on this processing level can be evaluated with temporal order judgment tasks and indexed by the individual temporal-order threshold value (TOT). This is defined as the shortest interval between two successive stimuli within a rapid sequence that is necessary for a listener to report their order with at least 75% correctness (Fink et al., 2005, 2006; Szymaszek et al., 2009, 2017; Szelag et al., 2011, 2018; Bao et al., 2013, 2014; Nowak et al., 2016; Jablonska et al., 2020).

Of course, shorter gaps (lower TOTs) correspond to higher temporal resolution and thus to better performance in the millisecond domain. Experimental studies, including previous studies conducted in our laboratory, have consistently indicated that the typical TOT in young healthy listeners is about 30–80 ms (Mills and Rollman, 1980; von Steinbüchel et al., 1999a,b; Szymaszek et al., 2009; Heinrich et al., 2014; Fostick and Babkoff, 2017). The data show also huge inter- and intra-individual variability in TOT. Some people are often not able to report temporal order correctly at shorter gaps, needing longer intervals between sequential stimuli. These people are characterized by lower temporal resolution. Interestingly, such lower resolution often corresponds with poorer cognitive functioning in comparison with those characterized by better resolution (Jablonska et al., 2020 in working memory; Nowak et al., 2016 in executive functions and Grondin et al., 2007; Oron et al., 2015; Szymaszek et al., 2017, 2018 in language capacity). Huge intra-individual differences in these cognitive functions have been frequently reported in previous studies and may be marked in every-day situations, as well as in various clinical samples (Szelag et al., 2004a,^2014^; Teixeira et al., 2013).

In the present study, temporal resolution in temporal order judgment was used to assess the participants’ efficiency in the sub-second range (see below).

#### Supra-Second Processing and Temporal Integration

It has been long known that the supra-second processing level plays an important role in cognitive functioning. The mental operations at this processing level are temporally segmented into intervals of a few seconds. Support for such temporal segmentation comes from observations of the temporal dynamics of fluent speech. In many languages, such as English, German, Polish, and Chinese, the semantic processing occurs in intervals of a few seconds and phrases (i.e., logical verbal segments) are limited in time to this duration (Pöppel, 1994, 1997). Such temporal chunking reflects the existence of a specific binding mechanism that links successive events (e.g., syllables or words) into longer perceptual units limited in time up to a few seconds.

Further support for the existence of such a processing platform comes from a number of literature studies concerning motor behavior, duration discrimination, reproduction and production of temporal intervals, sensorimotor-synchronization, spontaneous rate of change of perception of ambiguous figures, short term memory, slow cortical potentials, and mismatch negativity (for summary, see Pöppel, 1994, 1997, 2004, 2009; Szelag et al., 2002; Kowalska and Szelag, 2006; Matsuda et al., 2015; also Wang et al., 2015).

This body of evidence supports the thesis that the brain provides a temporal processing platform for our mental activity with a duration limited up to a few seconds. This platform may reflect the operation of the temporal integration mechanism—one of hypothetical mechanisms on the highest level of Pöppel’s framework model of time perception (see above). This mechanism binds sequences of elementary events together into perceptual (or conceptual) units of approx. 3 s durations. The existence of a temporal limit of a few seconds has been discussed for a long time in the literature and referred as the impression of the “subjective present” or the feeling of “now” (e.g., James, 1890; Fraisse, 1984; Pöppel, 1997, 2004; Dorato and Wittmann, 2020).

The limits of temporal integration across a time window of a few seconds may be also examined with the subjective accentuation paradigm (Szelag et al., 1996, 1997, 1998, 2004b; Szelag, 1997). This is the phenomenon reflecting that identical sounds within isochronous sequences can be perceived as unequal. In this paradigm, a listener hears a sequence of identical beats at one rate and, during such listening, by placing subjective accents on every n-th beat, the listener imposes a new rate, creating an individual rhythmic pattern. The listener can impose a new subjective structure onto identical sounds, but with a specific restriction: if the beats follow each other with an inter-beat-interval of, for example, 1 s, it is easy to impose a subjective structure by giving a subjective accent to every second or third beat; however, if the inter-beat-interval becomes too long (e.g., 5 s), the listener can no longer impose such a subjective structure and reports only separate beats (Szelag et al., 1997, 2004b). In such a case, temporal binding is impossible because the successive beats cannot be grouped within the time window of a hypothetical “subjective present,” as the integration would exceed the assumed 3 s duration.

Referring to the temporal segmentation of behavior mentioned above, the time period of around 3 s constitutes the fundamental unit related to the neuro-cognitive machinery in normal humans. Within such time frame information can be grasped as a unit, therefore, the longer integration indicates more effective processing because more events are linked together and processed as a *gestalt*.

Individual differences in the duration of such integration period were evidenced in our previous studies (Szelag et al., 2004b) indicating reduced binding in patients with receptive language problems in auditory comprehension. As in the normal sample the upper limit of integration corresponds to the typical duration of phrases lasting in the conversational speech a few seconds (Pöppel, 1994, 1997; Szelag et al., 2004b), the reduced integration reflects a situation where the listener’s brain cannot hold the information until the phrase is completed by a speaker. As a consequence, the ending of listener’s integration does not correspond to the ending of phrases produced by a speaker. The former seems too short to grasp the whole phrase as a unit causing comprehension problems. By analogy, reduced capacity of binding may accompany non-optimal cognitive functioning, for example, less efficient working memory in normal subjects.

Taking the above rationale into account, the subjective accentuation paradigm was also applied in the present study to measure efficiency in the supra-second domain (see below).

### Cross-Domains Relations in Temporal Information Processing

The above framework model raises the question of relationships between the different timescales. Given the evidence that our brains can generate discrete time quanta in the aforementioned two domains, there is ongoing debate as to whether performance on sub- and supra-second processing levels is related or whether they work independently in controlling behavioral activity (Mauk and Buonomano, 2004). In other words, the question is: are these two levels controlled by one underlying mechanism or by independent processes?

One may assume that such cross-domain overlapping may be expected from the theoretical point of view. Referring to the above hierarchical model of time perception, the rules of each hierarchy assume that each higher level should include phenomena observed at the lower level, but on the higher level new properties should be added.

The majority of studies on cross-domain comparisons focus on processing physical standards of defined durations in the sub-second range (typically up to 1 s, sometimes even up to 2 s) and the supra-second range (typically above a few seconds). These studies indicate a dissociation between these two timescales, suggesting the involvement of different neuronal processes in these two domains which are controlled by different mechanisms (e.g., Lewis and Miall, 2003a,b; Ulbrich et al., 2007; Morillon et al., 2009; Bangert et al., 2011; Gilaie-Dotan et al., 2011, 2016). Specifically, the sub-second range is assumed to be associated with motor and sensory processes and is known as “automatic timing.” In contrast, the supra-second range, known as “cognitive timing,” is associated more with cognitive mechanisms allowing the perception of accumulating durations.

The dissociation between sub- and supra-second interval timing is assumed to be reflected in the activity of neuronal circuits and represented by different types of oscillators generating spikes at regular intervals, which are built inside various circuits of the brain, known as “neural temporal units” (Merchant et al., 2008; Gupta, 2014; Gupta and Chen, 2016; Gupta and Merchant, 2017). This hypothesis is supported by the observation that training in discrimination between two durations can be generalized to different modalities but not to different durations (Buonomano and Karmarkar, 2002). As separate active clocks (pacemaker neurons) are proposed for each neural circuit, multiple calibration mechanisms of the proposed modular clock mechanism would be necessary to coordinate sub- and supra-second interval timing for controlling stable temporality and the impression of a continuous world (Gupta, 2014; Gupta and Chen, 2016).

Nevertheless, the studies mentioned above concentrated mostly on representation of the physical time of the perceived duration and, then, the encoding of the presented durations by neural circuits. These studies have predominantly employed duration judgment paradigms (reproduction, estimation, discrimination, comparison, etc.). In this context, one may ask about cross-domains interactions in perceptual timing for paradigms free from any duration judgment and, thus, from any translation of the physical time into neural processes. We therefore used such tasks in our study.

On the other hand, several various complementary models of TIP were proposed by Buonomano and Karmarkar (2002) supporting the idea of a centralized timing mechanism (or a pacemaker) for different tasks in parallel to separate populations of neurons for different intervals. Accordingly, in such labeled-line models, different intervals are coded by activity in independent and discrete populations of neurons. On the contrary, in population clock models, time is coded by the population activity of a large group of neurons and timing requires dynamic interaction between neurons for the parallel processing of interval, duration, order, and sequence cues. According to these authors, population models seem more likely to be the basis of timing in the range of tens to hundreds of milliseconds.

Convincing evidence for the neural representation of a pacemaker comes from electrophysiological and neuroimaging studies. Using event-related potential technology, Zhang et al. (2021) revealed similar activity patterns for sub- and supra-second time perception. Furthermore, Nani et al. (2019) provided a more recent meta-analysis of 84 published articles for a total of 109 experiments employing motor and non-motor (perceptual) tasks. They showed that both sub- and supra-second conditions recruit cortical and subcortical areas, but subcortical ones are activated more in sub-second tasks than those in supra-second tasks, in which a greater contribution from cortical activation was evidenced. However, in all studied conditions, common activations were observed in the SMA (rostral and caudal parts) along with the striatum and claustrum. These areas are supposed to be an essential node in different networks engaged in time processing.

To summarize, in light of this evidence, the mechanisms underlying cross-domain overlapping remain unclear and the question of relations between the timescales is still unanswered.

### Experimental Aim

Given the unclear relationships between TIP on sub- and supra-second levels, the present study asks whether we are equipped with a hypothetical core timing mechanism. Therefore we investigated whether better efficiency on the millisecond scale is accompanied by better processing on supra-second one. Specifically, we verified whether persons characterized by more efficient TIP in the sub-second domain considered as the basic level in the above framework hierarchical model are also more efficient in the supra-second domain. To avoid the above reservations regarding duration judgment paradigms which employ often the involvement of specific reference system for the use of conventional time units (e.g., seconds, Kowalska and Szelag, 2006), we used a novel approach—rarely studied before, as far as we are aware—employing for cross-domain comparisons the efficiency of temporal resolution in a temporal order judgment task (sub-second domain) and the limits of temporal integration in a subjective accentuation task (supra-second domain). These two paradigms incorporate the intrinsic TIP, free from any influences of the translation from physical time to neural processes.

## Materials and Methods

### Participants

The initial sample consisted of 118 young healthy volunteers (61 female/57 male), aged between 20 and 27 years (M ± SD = 23 ± 2 years). They were recruited *via* social media in the Warsaw area. All participants were right-handed native Polish speakers. They reported no systemic diseases, neurological or psychiatric disorders, head injuries in the past, addictions, or the use of medication that affects the nervous system. Moreover, all participants reported a lack of any regular musical education. Participants were screened for normal levels of cognitive abilities with the Raven Standard Progressive Matrices, as well as for normal hearing levels using pure-tone audiometry (Audiometer MA33, MAICO).

The study was in line with the Declaration of Helsinki and was approved by the Bioethics Committee of Nicolaus Copernicus University (permission no. KB 289/2019). All participants provided written informed consent prior to the study.

### Procedure

The experimental studies were conducted in a soundproof room in the Laboratory of Neuropsychology at the Nencki Institute of Experimental Biology. The methods included two parts: (1) screening for efficiency of sub-second timing using an auditory temporal-order judgment task and (2) assessment of efficiency of supra-second timing using a subjective accentuation task.

The experimental methods applied in both these paradigms were similar to those reported in our earlier papers; therefore they are only briefly summarized below. For the method applied in Part 1 see, for example, Szymaszek et al. (2009), Szelag et al. (2011, 2018), Bao et al. (2014), Nowak et al. (2016) for the method used in Part 2 see, for example, Szelag et al. (1996, 1997, 1998, 2004b), or Szelag (1997).

#### Part 1: Screening for Efficiency of Sub-Second Timing Using the Auditory Temporal-Order Judgment Paradigm

Two complementary tasks were applied using spatial and spectral stimulus presentation modes.

##### Stimuli

In both spatial and spectral tasks, paired acoustic stimuli were presented in rapid succession with various Inter-Stimulus Intervals (ISIs) separating the two stimuli in each pair. In the spatial presentation mode, paired clicks (square-wave pulses of 1 ms duration each) were presented monaurally in an alternating stimulation mode: one click was presented to one ear followed by another click to the other ear. In the spectral presentation mode, pairs of two 10 ms sinusoidal tones (i.e., a low tone of 400 Hz and a high tone of 3,000 Hz) were presented. These two paired tones were adjusted to equal loudness on the basis of isophones. The binaural stimulus presentation mode was used and each tone pair was presented to both ears.

The stimuli were generated by a computer with a sound controller using Waves MaxxAudio Pro software and presented *via* headphones at a comfortable listening level. The ISIs within each pair reflected the time gap between the offset of the first stimulus and the onset of the second stimulus. The duration of the ISIs varied during the experiment according to a pre-defined adaptive algorithm (see below for detailed description).

##### Tasks

**FIGURE 1:**
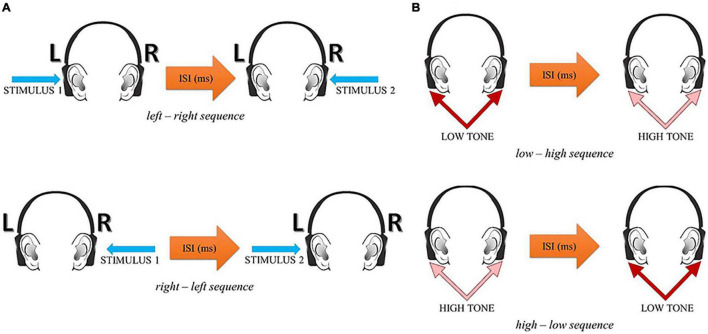
The experimental situation in the spatial **(A)** and spectral **(B)** temporal-order judgment tasks (reprinted from Szelag et al., 2018, p. 4).

The participant’s task was to report verbally the temporal order of two successive stimuli within each pair—their before—after relation. In the spatial mode, two alternative responses were possible: left–right or right–left. In the spectral mode, two alternative responses were possible: low–high or high–low. The experimental situation in these two tasks is displayed in Figure 1.

To control the duration of ISIs in consecutive trials, an adaptive algorithm based on maximum likelihood estimation was used. The implementation of this algorithm for testing young healthy listeners was based on the literature reports by Treutwein (1997), Fink et al. (2005, 2006), as well as on our previous studies (Wittmann and Szelag, 2003; Szymaszek et al., 2009; Szelag et al., 2011, 2018; Bao et al., 2013, 2014; Nowak et al., 2016).

The algorithm consisted of two steps (see Szelag et al., 2018, p. 4–5). In Step 1, the participant responded to 20 introductory trials comprising paired stimuli presented with fixed ISIs of varying durations in consecutive trials. They were presented first in decreasing (10 trials) and, subsequently, in increasing order (in the next 10 trials; i.e., down and up) according to pre-defined rules. In the spatial task, the ISI ranged from 160 to 1 ms (changing in 18 ms steps) and in the spectral task from 240 to 1 ms (changing in steps of 27 ms). The different testing ranges in the spatial and spectral tasks were based on our previous observations, indicating different performance in these two tasks in young participants. After completion of these 20 introductory trials, based on the correctness of the participant’s responses, the program calculated the ISI value for the initial trial in Step 2 of testing at the 75% probability of correct responses according to maximum likelihood estimation (Treutwein, 1997).

In Step 2, 50 trials were presented. In each of these 50 trials, the ISI was adjusted adaptively: it decreased after each correct response and increased after each incorrect response. The exact values of decreased or increased ISIs were randomly selected from a pre-defined range which varied depending on the tested ISI. To ensure accurate assessment, decrement steps were 0.5–5% of the ISI value of the previous trial, while increments were 10–20% of the previous ISI value.

On the basis of 70 completed trials (i.e., 20 trials in Step 1 and 50 trials in Step 2), the auditory Temporal Order Threshold (TOT) value for each participant was obtained as the mean of the estimated likelihood, calculated at 75% probability of correct responses (Treutwein, 1997). The measured TOT was defined as the shortest ISI between two successive stimuli necessary for a participant to report their temporal order with at least 75% correctness.

To focus the participant’s attention, each pair of stimuli was preceded by a warning signal delivered binaurally 1 s before the first stimulus in each pair. Then, the paired stimuli were presented monaurally (in the spatial task) or binaurally (spectral task). After each presentation, participants reported verbally the order of the two stimuli in the presented pair, i.e., left-right or right-left in the spatial task and high-low or low-high in the spectral task.

Prior to the collection of data, each participant was given a verbal instruction by the experimenter and, then, was presented with a few practice trials consisting of pairs with a relatively long ISI. In these practice trials, feedback on correctness achieved was given after each answer. All participants performed these practice trials satisfactorily. Next, the proper measurement started and no feedback on correctness was given.

The measurement was conducted with each participant individually in two separate sessions, separated by a break of a few days. In each session, both the spatial and spectral tasks were completed. The TOJ measurement lasted approximately 15 min for each task. The TOT values obtained in these two sessions were averaged and the mean TOT values in the spatial and spectral task were further analyzed.

##### Outcome Measure

The outcome measures were TOT values for the spatial and spectral tasks. These values reflected the participant’s TIP efficiency in the millisecond domain (sub-second level) in these two tasks. Accordingly, lower TOT values reflected better performance (HTE), whereas higher TOT values corresponded to poorer performance (LTE).

#### Part 2: Assessment of Efficiency in Supra-Second Timing Using a Subjective Accentuation Paradigm

##### Stimuli

The auditory stimuli were metronome beats (square-wave clicks of 1 ms duration) generated by the Adobe Audition 3.0 program and presented *via* earphones binaurally at the nine following frequencies: 1, 1.5, 2, 2.5, 3, 3.5, 4, 4.5, and 5 beats/s. This means that the inter-beat intervals in these sequences were: 1000, 667, 500, 400, 333, 286, 250, 222, and 200 ms, respectively. These frequencies of metronome beats were selected on the basis of our pretesting data, which showed that the majority of tested participants could not perform the experimental task when the beat rate was above or below the values enumerated above (see section “Introduction” for further explanation).

##### Task

**FIGURE 2:**
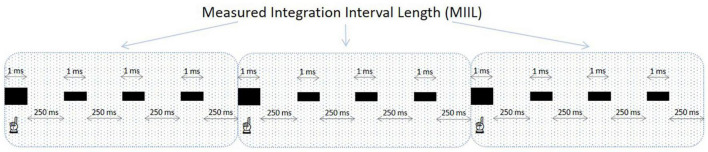
An example of a grouping of beats in the subjective accentuation task. The frequency of presented beats is 4 beats/s and the participant accentuates every fourth beat (the accentuated beats are bolded and indicated). In this sequence, the inter-beat interval is 250 ms and the MIIL is 1,000 ms (4 ms × 250 ms). When calculating the MIIL, the duration of the integrated beats (4 ms × 1 ms) is not included.

Participants were asked to listen to these equally spaced sequences of beats and to mentally accentuate during such listening every n-th (e.g., second, third, fourth, or other) beat to create an individual rhythmic pattern, integrating as many beats as possible. Obviously, this subjective rhythm existed only in the participant’s mind, but not objectively, as the presented sequences were monotonous in their nature and without any actual accentuation. After presentation, participants reported verbally the maximum number of beats they could unite into a rhythmic pattern for each presented beat sequence. This new group consisted of the accentuated beat and the unaccentuated ones following it. The time interval in which the participant could integrate the information was reflected in measured integration interval length (MIIL) obtained by multiplying the number of reported beats being united by a time distance between two successive beats at a given metronome ratio. The duration of MIIL corresponded to the upper limit of integration capacity. The experimental situation for the subjective accentuation task is displayed in Figure 2.

Prior to collection of the data proper, each participant was given a verbal instruction by the experimenter and, then, was presented with a few practice trials in which some examples of metronome sequences were played. All participants could unite beats during these example sequences and completed the practice trials satisfactorily. Next, the measurement proper started.

During the measurement proper, nine metronome frequencies (enumerated above) were presented 10 times each in random order. The MIIL values obtained for these 10 presentations were averaged and the mean MIIL for a given metronome frequency was further analyzed for each participant. The study comprised 90 trials. The measurement was conducted with each participant individually and lasted approximately 30 min.

##### Outcome Measure

The MIIL (in ms) calculated for particular metronome frequencies, reflecting the duration of perceptual unit comprising subjectively grouped beats (Figure 2). This MIIL was defined as the extent of temporal integration in supra-second time domain.

## Results

All statistical analyses were conducted using IBM^®^ SPSS^®^ Statistics 28.

### Study Design

The initial sample was screened for the level of temporal efficiency in the sub-second time domain using spatial and spectral temporal-order judgment tasks, which measured in each participant the temporal resolution in the auditory perception of temporal order. On the basis of the screening data obtained, two groups of participants were selected from the initial sample (*N* = 118): one group characterized by high temporal efficiency (HTE; *n* = 41) and the other group characterized by low temporal efficiency (LTE; *n* = 40). See below for the detailed procedure used to classify participants and the characteristics of these two groups. Next, the efficiency of performance of the HTE and LTE groups in the supra-second domain was compared using the subjective accentuation task. Finally, the relationships between the efficiency of TIP in sub- and supra-second ranges were tested in the HTE and LTE in the spatial and spectral task separately using the Spearman’s rank correlation analysis.

### Classification of Participants According to Their Efficiency in Sub-Second Timing

**FIGURE 3:**
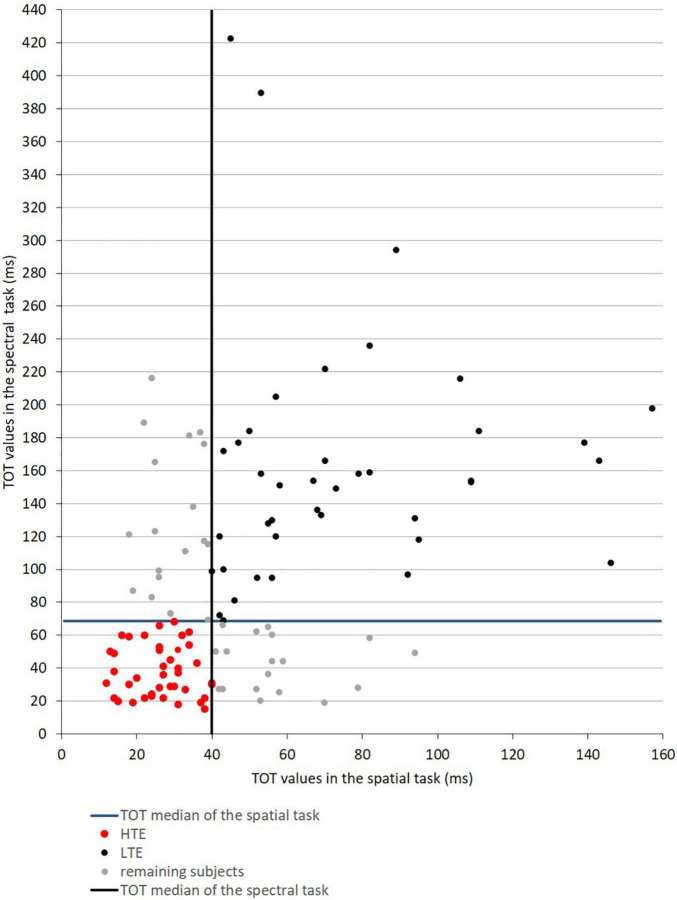
Scatter plot data presenting TOT values obtained in the spatial and spectral temporal-order judgment tasks in the initial participant sample (*N* = 118). The vertical and horizontal solid lines reflect the median values of TOT in these two tasks, indicating four quartiles of obtained results depicted with different colors.

The median TOT values obtained in the initial participant sample (*N* = 118) in the spatial task was 40 ms and in the spectral task was 69 ms. On the basis of these two median values, two groups of participants (namely HTE and LTE) were selected from the initial sample (for the selection procedure, see Figure 3). These two far groups were selected to compare the performance between the defined efficiency of TIP, considering the clear-cut points between HTE and LTE. The remaining participants—those characterized by medium TOT values—were not considered in further analyses.

The HTE Group (*n* = 41, marked with red dots) was characterized by TOT values in both tasks below the median TOT. The LTE Group (*n* = 40, black dots) was characterized in both tasks by TOT values above the median TOT. Next, in the HTE and LTE groups the mean TOT in each task was calculated and further analyzed. The remaining participants (*n* = 37, gray dots) indicated mixed temporal efficiency—below the median TOT in one task but above this median TOT in the other, or vice versa. Therefore, they were not considered in further analyses. Detailed characteristics of the HTE and LTE groups are given in Table 1.

**TABLE 1:** Characteristics of groups.

Group	*n*	Age (years)		Sex	TOT (mean)	
		Range	Mean (SD)	(Female/male)	Spatial	Spectral
HTE	41	From 20 to 27	23 (2)	16/25	26 ms	38 ms
LTE	40	From 20 to 26	23 (2)	23/17	75 ms	162 ms

This classification of participants into HTE and LTE groups was further confirmed in two separate 2-way analyses of variance (ANOVAs) conducted on TOT values from the spatial (ANOVA 1) and spectral (ANOVA 2) tasks with “Group” (HTE vs. LTE) as between-subject variable. The data submitted to these analyses were transformed by natural logarithm, because the distribution of TOT values obtained in the spectral task deviated from the Gaussian distribution.

These two analyses revealed only a significant effect of “Group” [*F*(1, 79) = 89.09; *p* < 0.001; η2 = 0.53 and *F*(1, 79) = 255.8; *p* < 0.001; η2 = 0.76, for spatial and spectral tasks, respectively]. In the HTE group, the mean TOT value (26 ms for the spatial task and 38 ms for the spectral task) was lower than in the LTE group (75 ms for the spatial task and 162 ms for the spectral task), indicating better performance in the HTE group.

### Supra-Second Timing Efficiency: Subjective Accentuation Task

**FIGURE 4:**
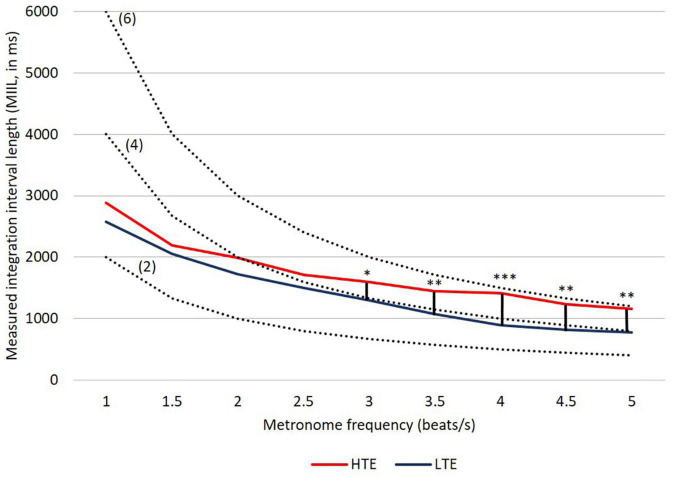
MIILs for various metronome tempos in HTE and LTE groups. Gray lines reflect two hypothetical integration strategies: (A) integration in a constant time limited up to e.g., 3,000, 2,500, or 2,000 ms (solid horizontal lines) and (B) integration by mental counting ignoring the constant time (e.g., up to 2, 4 or 6 beats, dashed lines). Significant differences between groups are marked with asterisks: *p < 0.05; ^**^p < 0.01; ^***^p < 0.001. For more details see Table 2 and text below.

The results of the present study are congruent, in general, with our previous observations using the subjective accentuation paradigm (Szelag et al., 1996, 1997, 2004b), indicating a specific integration process in supra-second intervals. The young healthy volunteers studied here could bind mentally temporally separated successive beats into larger perceptual units independently of their efficiency in sub-second timing (Figure 4). Thus, the separated beats were organized at the perceptual level into a higher-order structure that dominated their serial order.

**TABLE 2:** The MIIL for particular metronome frequencies in HTE and LTE groups.

Group	Presented metronome frequencies (beats/s)								
	1	1.5	2	2.5	3 *	3.5 **	4 ***	4.5 **	5 **
HTE	2,880	2,193	1,990	1,713	**1,599**	**1,446**	**1,412**	**1,237**	**1,156**
LTE	2,573	2,055	1,719	1,500	**1,297**	**1,073**	**888**	**818**	**779**

Significant differences between groups (bolded) are marked by asterisks: *p < 0.05; **p < 0.01; ***p < 0.001.

Similarly to our previous studies, the MIIL in both groups strongly depended on the presented metronome frequency, but some regularities observed previously were also confirmed (Figure 4). Namely, for the lowest metronome frequency (1 beat/s) the MIIL did not exceed the 3 s time window which is typically assumed as the maximum limit of the temporal integration mechanism (Pöppel, 2004, 2009). On the other hand, for higher frequencies, the duration of MIIL was systematically shortened up to about 1 s.

Looking at the Figure 4, one can infer that at least three different strategies were used in the subjective accentuation task. First, if participants integrate information only by time (i.e., in a constant period of, e.g., 2, 3, or 4 s), the MIIL would be constant and independent of the presented frequency (horizontal continuous lines, Figure 4). Second, if the participants integrate information only by number (i.e., counting a constant number of beats, e.g., 2, 3, or 4), the MIIL would depend strongly on the presented frequency (dashed lines, Figure 4). Third, a combination of these two strategies would be possible in both groups, but integration by constant time dominates in the HTE group, whereas, in contrast, the LTE group supported the integration process with mental counting, especially for the higher metronome ratios.

To compare the performance of the HTE and LTE groups, the MIIL (transformed by square root extraction) values were submitted to a 2-way analysis of variance (ANOVA). The design included “Group” (HTE vs. LTE) as the between-subject variable and “Metronome Frequency” (nine ratios: 1, 1.5, 2, 2.5, 3, 3.5, 4, 4.5, and 5 beats/s) as a within-subject variable. Greenhouse-Geisser correction was applied on the basis of results of Mauchly’s test for sphericity. After the analysis of variance, to determine the sources of significance, the Bonferroni *post hoc* procedure was applied.

The analysis yielded a significant main effect of “Group” [*F*(1, 79) = 8.58, *p* < 0.01, η2 = 0.10], “Metronome Frequency,” [*F*(2.53, 199.48) = 102.93, *p* < 0.01, η2 = 0.57], as well as the “Group × Metronome Frequency” interaction [*F*(2.53, 199.48) = 3.42, *p* = 0.03, η2 = 0.04].

In the HTE group, the mean MIIL (1,736 ms) was longer than that in the LTE group (1,411 ms). However, this main effect was modified by the presented metronome frequency. Pairwise comparison tests with Bonferroni correction showed that significant differences between groups were observed only for higher metronome frequencies (i.e., from 3 up to 5 beats/s), being non-significant for lower frequencies (i.e., from 1 up to 2.5 beats/s). These relationships are illustrated in Figure 4. For more descriptive statistics see Table 2.

These relationships between the efficiency of TIP in sub- and supra-second ranges in the HTE and LTE were further confirmed by the Spearman’s rank correlations. We observed significant negative correlations between the TOT values achieved in the temporal-order judgment task (in spatial and spectral tasks, separately) and MIILs obtained in subjective accentuation task (Table 3). Better temporal resolution (lower TOT values) was accompanied by longer integration (longer MIILs) for all metronome tempos with the exception of 1.5 and 2 beats/s.

**TABLE 3:** Spearman’s rho correlation coefficients (and significance levels) between TOT values obtained in the spatial and spectral temporal-order judgment task and MIILs for various metronome tempos.

TOT task	Presented metronome frequencies (beats/s)								
	1	1.5	2	2.5	3	3.5	4	4.5	5
Spatial	−0.234*	−0.161	−0.208	−0.226*	−0.219*	−0.284*	−0.315**	−0.242*	−0.246*
Spectral	−0.242*	−0.128	−0.168	−0.217^#^	−0.239*	−0.322**	−0.370**	−0.266*	−0.271*

Asterisks indicate significant correlations: *p < 0.05; **p < 0.01; ^ #^p < 0.051.

## Discussion

The results of the two experiments presented here provide convincing evidence for the existence of a close relationship between the efficiency of TIP in sub- and supra-second ranges. The clear relationships were evident for two different indices of participants’ performance: TOT in the temporal-order judgment task (sub-second level) and MIIL in the subjective accentuation task (supra-second level). It is important to emphasize that these two tasks employed totally different experimental procedures based on intrinsic timing operations to measure performance on these two levels.

It is interesting to note that listeners characterized by more efficient temporal resolution (i.e., HTE) could integrate information in the supra-second domain in longer units, reflecting more efficient temporal integration. In contrast, subjects less efficient in such resolution (i.e., LTE) indicated, in parallel, less efficient integration reflected in shorter MIILs than those observed in HTE (Figure 4 and Table 2). Despite these differences, in both groups the upper limit of integration evidenced in the subjective accentuation task was within 3 s time “window.”

Another important result was the use of different integration strategies in the LTE and HTE groups; however, this was evidenced only in situations in which the processed material required higher mental load—for higher metronome ratios (from 3 up to 5 beats/s; Figure 4 and Table 2). In these situations, LTE relied more on the mental counting of consecutive beats (usually up to 4), but not so much on integration based on constant time related to the limits of temporal integration, as used by HTE in these situations (Figure 4). The application of such a counting strategy in LTE resulted in the creation of shorter rhythmic patterns (shorter MIIL), in contrast to the longer patterns in HTE, reflecting the ability to keep the information in longer units. The information processing in the latter group seems more efficient than the former one (see section “Introduction” for more explanations). The cross-domain relations in HTE and LTE reported here were further confirmed by the negative correlations, indicating that better temporal resolution (lower TOT values) was accompanied by longer binding (longer duration of MIILs) for nearly all metronome tempos (Table 3). To sum up, LTE displayed narrowed binding resources within a few seconds because of a shorter (i.e., less efficient) span of integration resources in tasks with higher mental load, as exemplified by the higher metronome frequencies presented here.

It should be stressed that, for such integration capacity, the difference between HTE and LTE was non-significant for tasks with lower mental load, exemplified by lower metronome ratios (i.e., from 1 up to 2.5 beats/s; Figure 4 and Table 2). This means that the upper limit of temporal integration remained relatively stable, independently of the temporal resolution power. In both HTE and LTE, a similar integration strategy was employed for lower metronome ratios, as these two groups relied partially on constant time and partially on mental counting (Figure 4). Finally, our results support the thesis that the upper limit rarely exceeded 3 s intervals and was resistant to temporal resolution power, indexed by TOT.

The cross-domain relations reported in these two paradigms support the thesis that one “clock” (or neural mechanism) may be used for sub- and supra-second tasks. A solid conceptual background for understanding these relations may be provided by once again referring to Pöppel’s taxonomy of TIP—in particular, to temporally discrete information processing within a time window of approx. some tens of milliseconds (see section “Introduction”). Despite important individual differences, much experimental data has indicated that the perception of succession is controlled by the central timing mechanism, probably implemented in neuronal oscillations with a periodicity of about 25–40 Hz observed in electrophysiological activity (Van Rullen and Koch, 2003; Fink et al., 2006; Bao et al., 2013). Each period of such oscillations reflects an elementary processing unit (a system state) of a duration of approx. 30 ms. At a theoretical level, therefore, it was hypothesized that two events occurring within one such system state will be treated as co-temporal and fused into one unit. As a consequence, the before–after relationship cannot be established. In contrast, the before–after relation can be perceived if two stimuli occur in at least two successive oscillatory periods (Pöppel, 2009). Considerable data supporting the thesis of a common basic mechanism underlying the perception of temporal order can be provided from experiments on different sense modalities, indicating that TOT appears to have a similar numerical value of some tens of milliseconds for different sense modalities (visual, auditory, tactile; e.g., Hirsh and Sherrick, 1961; Pöppel, 1994, 1997; Kanabus et al., 2002).

The question remains as to what neural process could be a potential source of such oscillatory activity, providing the temporal constraints for sequencing ability? There is strong evidence that spontaneous (or stimulus triggered) gamma band oscillations corresponding in periodicity to the value of TOT play an important role in human cognition (Poeppel, 2003; Van Rullen and Koch, 2003; Heim et al., 2013). Furthermore, one may expect that the periodicity of neuronal oscillations might be modified by a hypothetical pacemaker, resulting in lower or higher TOT. For example, a higher pacemaker rate might lead to shorter periods of gamma oscillations and lower TOT, as evidenced in the HTE group. Conversely, a lower rate in such a hypothetical pacemaker might lead to longer periodicity of such oscillations and higher TOT values, reflected in our study in less efficient sequencing abilities in the LTE group (Figure 3). This could provide a theoretical explanation of the problem of individual differences in temporal resolution power.

Another electrophysiological candidate for a timekeeping mechanism could also be the beta rhythm, with a periodicity of 14–30 Hz (see van Wassenhove et al., 2019 for a recent review). Recently, evidence for the contribution of the beta rhythm to timing behavior was found in synchronization–continuation tasks in primates (e.g., Bartolo and Merchant, 2015) as well as in humans in tasks addressing predominantly supra-second timing. Specifically, Kononowicz and van Rijn (2015) reported the contribution of the beta power to the production of temporal intervals of 2.5 s duration, Kulashekhar et al. (2016) in duration judgment, and Wiener et al. (2018) in lengthening of duration experienced subjectively using transcranial alternating current stimulation (tACS). Moreover, Bernasconi et al. (2011) provided evidence for the contribution of beta range oscillations (18–23 Hz) to the perception of order in temporal order judgment tasks. To sum up, we cannot exclude the hypothesis that beta rhythm oscillations of a frequency of approx. 14–30 Hz (i.e., one period of ca. 30–70 ms duration) contribute to a time keeping mechanism in sub-second TIP.

The converging lines of evidence briefly summarized above consistently indicate that auditory perception of temporal order may represent a very basic mechanism of information processing rooted in electrophysiological indices. Further studies are needed to clarify which rhythm can be considered to be a basic oscillator that may actuate the pacemaker of the hypothetical internal clock. However, there is no doubt that temporal resolution is represented endogenously and based on intrinsic neural operations. They seem relevant for many cognitive functions, in both normal states and in pathological conditions (Nowak et al., 2016; Jablonska et al., 2020). Of course, these operations may be influenced by the nature of the presented material (spectral vs. spatial), as well as stimulus presentation modes (monaural vs. binaural). These influences were discussed in detail in our previous paper (Szelag et al., 2018).

The cross-domain relationships summarized in the Introduction point to a dissociation between neural circuits involved in TIP in different timescales. An important problem may be that these comparisons have often focused on duration judgment methods, using various paradigms involving the translation of classical time units (usually seconds or milliseconds) into neural processes. Such translation processes are not necessarily the same for different timescales. It would be difficult to accept that the same time frame operates in sub- and supra-second scales. To avoid such reservations, in the present study we tested endogenous mechanisms involved in the perception of temporal order and in temporal integration, which seem to be free from any bias from the classic time units and are rooted in intrinsic timing operations. It is a critical question whether such cross-domain dissociation may be also present in the case of the endogenous operations investigated in our study.

### Final Conclusion

The results of the present study suggest that intrinsic timing operations on sub-second level may regulate TIP on the supra-second range. We can conclude that the temporal resolution in the tens of millisecond range reflected in the perception of temporal order is incorporated also at higher levels of TIP and may be essential for predicting individuals’ efficiency in binding operations at the supra-second domain. Thus, neural entrainment in the sub-second range may help the brain to calibrate its timing for information processing in the supra-second range.

## Data Availability Statement

The raw data supporting the conclusions of this article will be made available by the authors, without undue reservation.

## Ethics Statement

The studies involving human participants were reviewed and approved by Bioethics Committee of Nicolaus Copernicus University (Permission No. KB 289/2019). The patients/participants provided their written informed consent to participate in this study.

## Author Contributions

ES conceptualized and designed the study, interpreted the data, wrote the manuscript, and is responsible for the final version of the manuscript. MS recruited the participants, acquired, analyzed and interpreted the data, and wrote the manuscript. AS interpreted the data and wrote the manuscript. All authors approved the final version of the manuscript.

## Conflict of Interest

The authors declare that the research was conducted in the absence of any commercial or financial relationships that could be construed as a potential conflict of interest.

## Publisher’s Note

All claims expressed in this article are solely those of the authors and do not necessarily represent those of their affiliated organizations, or those of the publisher, the editors and the reviewers. Any product that may be evaluated in this article, or claim that may be made by its manufacturer, is not guaranteed or endorsed by the publisher.

## Abbreviations

TIP: temporal information processing
ISI: inter-stimulus-interval
TOT: temporal-order threshold
MIIL: measured integration interval length
HTE: high temporal efficiency
LTE: low temporal efficiency.
